# A novel algorithm for simultaneous SNP selection in high-dimensional genome-wide association studies

**DOI:** 10.1186/1471-2105-13-284

**Published:** 2012-10-31

**Authors:** Verena Zuber, A Pedro Duarte Silva, Korbinian Strimmer

**Affiliations:** 1 Institute for Medical Informatics, Statistics and Epidemiology, University of Leipzig, Härtelstr. 16–18, D-04107 Leipzig, Germany; 2 Faculdade de Economia e Gestão & CEGE, Catholic University of Portugal, Rua Diogo Botelho 1327, 4169-005 Porto, Portugal

## Abstract

**Background:**

Identification of causal SNPs in most genome wide association studies relies on approaches that consider each SNP individually. However, there is a strong correlation structure among SNPs that needs to be taken into account. Hence, increasingly modern computationally expensive regression methods are employed for SNP selection that consider all markers simultaneously and thus incorporate dependencies among SNPs.

**Results:**

We develop a novel multivariate algorithm for large scale SNP selection using CAR score regression, a promising new approach for prioritizing biomarkers. Specifically, we propose a computationally efficient procedure for shrinkage estimation of CAR scores from high-dimensional data. Subsequently, we conduct a comprehensive comparison study including five advanced regression approaches (boosting, lasso, NEG, MCP, and CAR score) and a univariate approach (marginal correlation) to determine the effectiveness in finding true causal SNPs.

**Conclusions:**

Simultaneous SNP selection is a challenging task. We demonstrate that our CAR score-based algorithm consistently outperforms all competing approaches, both uni- and multivariate, in terms of correctly recovered causal SNPs and SNP ranking. An R package implementing the approach as well as R code to reproduce the complete study presented here is available from http://strimmerlab.org/software/care/.

## Background

Genome-wide associations studies (GWAS) are now routinely conducted to search for genetic factors indicative of or even causally linked to disease. Typically, the aim of such a study is to identify a small subset of single nucleotide polymorphisms (SNPs) associated with a phenotype of interest. From an analysis point of view the screening for relevant biomarkers is best cast as a problem of statistical variable selection. In GWAS variable selection is very challenging as the full set of SNPs is often very large while both the effect of each potentially causal SNP as well as their number is very small (e.g. [1-3]).

To date, most GWAS are based on single-SNP analyzes where each SNP is considered independently of all others and association with the phenotype is computed using a univariate test statistic such as variants of the *t*-score, the ATT statistic [4] or marginal correlation [5]. The advantage of this approach is that it is computationally inexpensive. However, it implicitly assumes complete independence of markers and thus ignores the correlation structure among SNPs, e.g., due to linkage or interaction among SNPs.

In order to increase statistical efficiency and to exploit the correlation among predictive SNPs several authors have recently started to investigate simultaneous SNP selection using fully multivariate approaches. This was pioneered for GWAS in the seminal paper of [1] that introduced the NEG regression model, a shrinkage-based approach to select relevant SNPs. A related approach is LASSO regression that was employed to GWAS by [6], MCP regression [2], and Bayesian variable selection regression [3]. Another promising multivariate approach advocated for high-dimensional variable selection is boosting [7] but this has not yet been investigated for GWAS.

Recently, to address the problem of variable importance and selection under correlation in genomics, we have introduced two novel statistics, the correlation-adjusted *t*-score (CAT score) and the correlation-adjusted marginal correlation (CAR score), see [8,9]. These two measures are multivariate generalizations of the standard univariate test statistics that take the correlation among variables explicitly into account and lead to improved rankings of markers as has been shown for data from transcriptomics and metabolomics. However, application of CAT and CAR scores has so far been restricted to medium to large dimensional settings only as computing these scores involves the calculation of the inverse matrix square root of the correlation matrix, which is prohibitively expensive in high dimensions. Thus, for SNP analyzes further computational economies are needed.

Here, we develop a novel multivariate algorithm for large scale SNP selection using CAR score regression. Specifically, we propose a computationally efficient procedure that allows for shrinkage estimation of CAR scores even for very high-dimensional data sets. Subsequently, we conduct a systematic comparison of state-of-the-art simultaneous SNP selection procedures using data from the GAW17 consortium [10]. These data are particularly suited for investigating relative performance as the true causal SNPs are known. Finally, we demonstrate that SNP rankings based on correlation-adjusted statistics consistently outperform all investigated competing approaches, both uni- and multivariate.

## Methods

### Univariate ranking of SNPs

The basic setup we consider here is a linear regression model for a set of *d* predictors ***X ***= {*X*_1_,…,*X*_*d*_} and a metric or binary response variable *Y *. In GWAS the covariates ***X*** are given by the genotype and the response *Y * is the phenotype or trait of interest. The correlation matrix among the predicting variables has size *d *×* d* and is denoted by ***P*** (capital “rho”). The vector of marginal correlations ***P***_*XY *_= (*ρX*_1_*Y*,…,*ρX*_*d*_*Y*)^*T*^contains the correlations between a metric response and each individual SNP. Similarly, for binary response the *t*-score vector ***τ ***= (*τ*_1_,…,*τ*_*d*_)^*T*^contains the *t*-scores computed for each variable.

If there is no correlation among SNPs (i.e. ***P ***=*** I***_*d*_) the *t*-scores ***τ ***provide an optimal ranking of SNPs in terms of predicting a binary *Y *[11]. Likewise, for metric response the marginal correlations lead to an optimal ordering [12]. Moreover, in the absence of SNP-SNP correlation the squared values of the ranking statistics (squared *t*-score, squared marginal correlation) are useful measures of variable importance, adding up to Hotelling’s *T*^2^ and the squared multiple correlation coefficient *R*^2^, respectively.

### CAT and CAR score

In many important settings the correlations ***P ***do not vanish but rather represent additional structure relating the predictors. In the case of SNPs the correlation may be rather large, e.g. due to linkage effects [13]. Thus, both for variable ranking and for assigning variable importance it can be essential to take the correlation between covariates into account.

To this end we have proposed a simple modification of the *t*-statistic and marginal correlations. In [8] we have introduced the CAT score (correlation-adjusted *t*-score) that is defined as

(1) $$\tau^{\text{adj}}=P^{-1/2}\tau$$

where ***P***^−1/2^ is the inverse of the matrix square-root of ***P***. The vector ***τ***^adj ^contains the adjusted *t*-scores which measure the influence of each predictor on *Y * after simultaneously removing the effect of all other variables. The squared CAT score may thus be used as measure of variable importance. Unlike squared *t*-scores they sum up to Hotelling’s *T*^2^ even in the presence of correlation,

$${\left(\tau^{\text{adj}}\right)}^{T}\tau^{\text{adj}}=\tau^{T}P^{-1}\tau =T^{2}.$$

Correspondingly, in [9] we investigated a correlation-adjusted marginal correlations (CAR scores)

(2) $$P_{XY}^{\text{adj}}=P^{-1/2}P_{XY}\;.$$

The squared CAR scores sum up to the squared multiple correlation coefficient

$${\left(P_{XY}^{\text{adj}}\right)}^{T}P_{XY}^{\text{adj}}=P_{YX}P^{-1}P_{XY}=R^{2}\;,$$

also known as coefficient of determination or proportion of variance explained. Because of this decomposition property CAT and CAR scores allow to assign importance not only to individual SNPs but also to groups of SNPs. Moreover, both CAT and CAR score share a grouping property that leads to similar scores for highly correlated SNPs. In addition they protect against antagonistic SNPs, i.e. if two SNPs are highly correlated and one has a protective and the other a risk effect, then both SNPs are assigned low scores.

For model selection using CAT and CAR scores, i.e. for identification of those SNPs that do not contribute to predict the response *Y *, we use a simple thresholding procedure with the critical threshold obtained by controlling local false discovery rates [14].

In previous work we have shown for synthetic data as well as for data from metabolomic and gene expression experiments that CAT and CAR scores are effective multivariate criteria for obtaining compact yet highly predictive feature sets. Independently, in the study of [15] it was also found that CAT scores result in favorable orderings of variables.

However, with increasing dimension *d* the correlation matrix ***P*** becomes prohibitively large both to compute and to handle effectively. As a result, in high dimensions direct calculation of CAT and CAR scores using Eq. 1 and Eq. 2 is not possible. Thus, for application in high-dimensional data such as from GWAS an alternative means of computation must be developed.

### Computationally efficient calculation of shrinkage estimators of CAT and CAR scores

If the number of observations *n* is smaller than the number of variables *d* we need to employ a regularized estimate for the correlation matrix ***P***. A simple shrinkage estimator ***R ***for ***P*** is given by

$$R=\lambda I_{d}+(1-\lambda )R_{\text{empirical}}$$

where ***R***_empirical_ is the empirical non-regularized correlation matrix and *λ *is a shrinkage intensity (e.g. [16]). Using computational economies akin to those discussed in [17] we now show that computation of ***R***^−1/2 ^and subsequent calculation of estimates of CAT and CAR scores can be done in a computationally highly effective way, even when direct computation of CAT and CAR scores via Eq. 1 and Eq. 2 is infeasible.

Using singular value decomposition the empirical correlation matrix can be written ***R***_empirical _=* λ*/(1−*λ*)***UMU***^*T*^ where ***M*** is positive definite matrix of size *m *×* m*, ***U*** an orthonormal matrix of size *d *×* m*, and *m *= rank(***R***_empirical_) <<* d*. This simplifies the shrinkage estimator to

$$R=\lambda (I_{d}+UMU^{T})\;.$$

Following [8] we then compute the *α*-th matrix power of ***R ***using

$$R^{\alpha }=\lambda^{\alpha }(I_{d}-\underset{d\times m}{\underset{︸}{U}}(I_{m}-{\left(\underset{m\times m}{\underset{︸}{I_{m}+M}}\right)}^{\alpha })\underset{m\times d}{\underset{︸}{U^{T}}})\;.$$

This implies we only have to compute the matrix power of the *m *×* m* matrix ***I***_*m*_ + ***M*** to obtain ***R***^*α*^. Moreover, for efficiently calculating CAT and CAR scores it is crucial to note that it is not at all necessary neither to store or to compute the full *d *×* d* sized matrix ***R***^−1/2 ^as

(3) $$\begin{array}{l} R_{XY}^{\text{adj}}=R^{-1/2}R_{XY}\\ \;=\lambda^{-1/2}(I_{d}-U(I_{m}-{\left(I_{m}+M\right)}^{-1/2})U^{T})R_{XY}\\ \;=\lambda^{-1/2}(\underset{d\times 1}{\underset{︸}{R_{XY}}}-\underset{d\times m}{\underset{︸}{U(I_{m}-{\left(I_{m}+M\right)}^{-1/2}}})(\underset{m\times 1}{\underset{︸}{U^{T}R_{XY}}}))\;. \end{array}$$

Consequently, Eq. 3 allows to obtain shrinkage estimates of CAT and CAR scores effectively even in high dimensions as none of the matrices employed in Eq. 3 is larger than *d *×* m*, and most are even smaller (*d *× 1 or *m *× 1), all without actually computing the shrinkage correlation matrix ***R***.

## Results and discussion

We now compare the proposed CAR score approach to simultaneous SNP selection with competing methods and determine its effectiveness in finding true causal SNPs.

For this purpose we use the mini-exome data set compiled for the GAW17 workshop held 13-16 October 2010 in Boston ( http://www.gaworkshop.org/gaw17/). This data set is a combination of real sequence data and simulated synthetic phenotypes, where the true causal SNPs are known. In our study we investigate univariate ranking by marginal correlation and five multivariate approaches.

In order to facilitate replication of our results we provide complete R code [18]. Our R package “care” implements the developed algorithm. Moreover, we offer R scripts covering all analysis steps from preprocessing the raw data to plotting of figures at http://strimmerlab.org/software/care/. The data are publicly available from the GAW consortium, see http://www.gaworkshop.org/gaw17/data.html for details.

### GAW 17 unrelated data

The compilation and simulation of phenotypes for the GAW17 mini-exome data set is described in detail in [10]. We focus here on the GAW 17 unrelated data with metric phenotypes Q1, Q2, and Q4. The corresponding sequence data matrix contains information on 24,487 SNPs for *n *= 697 individuals. For each phenotype there are *B *= 200 simulations. By construction, phenotype Q1 has a residual heritability of 0.44 and is influenced by 39 SNPs in 9 genes, whereas Q2 has a lower residual heritability of 0.29 and is influenced by 72 SNPs in 13 genes. This suggests that discovery of true causal SNPs should be less challenging for Q1 than for Q2. Phenotype Q4 has a heritability of 0.70 but none of it is due to SNPs contained in the present data set.

### Preprocessing

In the preprocessing of the sequences we first recoded the alleles in the raw data into 0, 1, 2 assuming an additive effects model. Second, we standardized the data matrix to column mean zero and column variance 1. Subsequently, we removed duplicate predictors so that 15,076 unique SNPs remained. The set of true causal SNPs for both Q1 and Q2 also contains each a duplicate, reducing the number of true unique SNPs to 38 and 71. Finally, we further filtered out synonymous SNPs, as we are interested only in non-synonymous mutations. The resulting predictor matrix ***X*** is of size 697 × 8,020, i.e. *d *= 8,020 unique non-synonymous SNPs are simultaneously considered for selection.

For preprocessing the response variables Q1, Q2, and Q4 we removed the influence of the three non-genetic covariates sex, age, and smoking by linear regression. The resulting residuals were standardized to mean zero and variance 1 which yielded *B *= 200 response vectors $y_{1}^{\left(b\right)}$, $y_{2}^{\left(b\right)}$, and $y_{4}^{\left(b\right)}$, where *b *∈ 1,…,*B*, each of size 697 × 1.

### SNP selection methods included in the comparison study

For each of the *B *= 200 response vectors for Q1, Q2, and Q4 we computed a regression model including all *d *= 8,020 SNPs as potential predictors. Following [2] we focused on regularized regression approaches. Specifically, we used the following five methods, all of which have been shown to be powerful tools for variable selection in large-scale regression settings: 

• CAR: variable ranking by shrinkage CAR scores [9],

• NEG: regression with normal exponential gamma (NEG) prior [1],

• MCP: regression with MCP penalty [19],

• BOOST: boosting [20], and

• LASSO: lasso regression [21].

The corresponding software implementations are listed in Table 1. As a reference for comparison we additionally included two baseline methods: 

• COR: univariate SNP ranking by marginal correlation, and

**Table 1:** Software used in the comparison study

**Method**	**Software**	**Reference**
CAR	R package care	[9]
COR	R package care	[9]
NEG	HLasso program	[1]
MCP	R package ncvreg	[22]
BOOST	R package mboost	[7]
LASSO	R package glmnet	[23]

The R packages are available from the R software archive CRAN at http://cran.r-project.org/.

RND: random ordering of all SNPs.

All methods except CAR and COR combine regularization with variable selection. Thus, for determining model sizes for CAR scores and COR we adaptively estimated a threshold from the data using a local FDR cutoff of 0.5 as recommended in [14]. In settings with rare and weak features this particular choice coincides with the so-called “higher criticism” threshold that has shown to be powerful for signal identification in classification (e.g., [24-26]). For computing the FDR values we employed the R package fdrtool[27,28].

Generally, all software were run with default settings. The regularization parameters required by the NEG, MCP, BOOST and CAR approaches were set to fixed values optimizing the overall performance of each method. Specifically, for CAR and MCP we employed *λ *= 0.1, for BOOST *ν *= 0.1, and for NEG *λ *= 85. For LASSO we used the built-in cross-validation routines.

### Relative performance of investigated methods

The aim of this study is to compare simultaneous SNP selection methods with regard to their ability to discover the true known SNPs. For this purpose we investigated the respective SNP rankings and the corresponding true positives, the size of the selected models, and the variability across the 200 repetitions.

**Figure 1:**
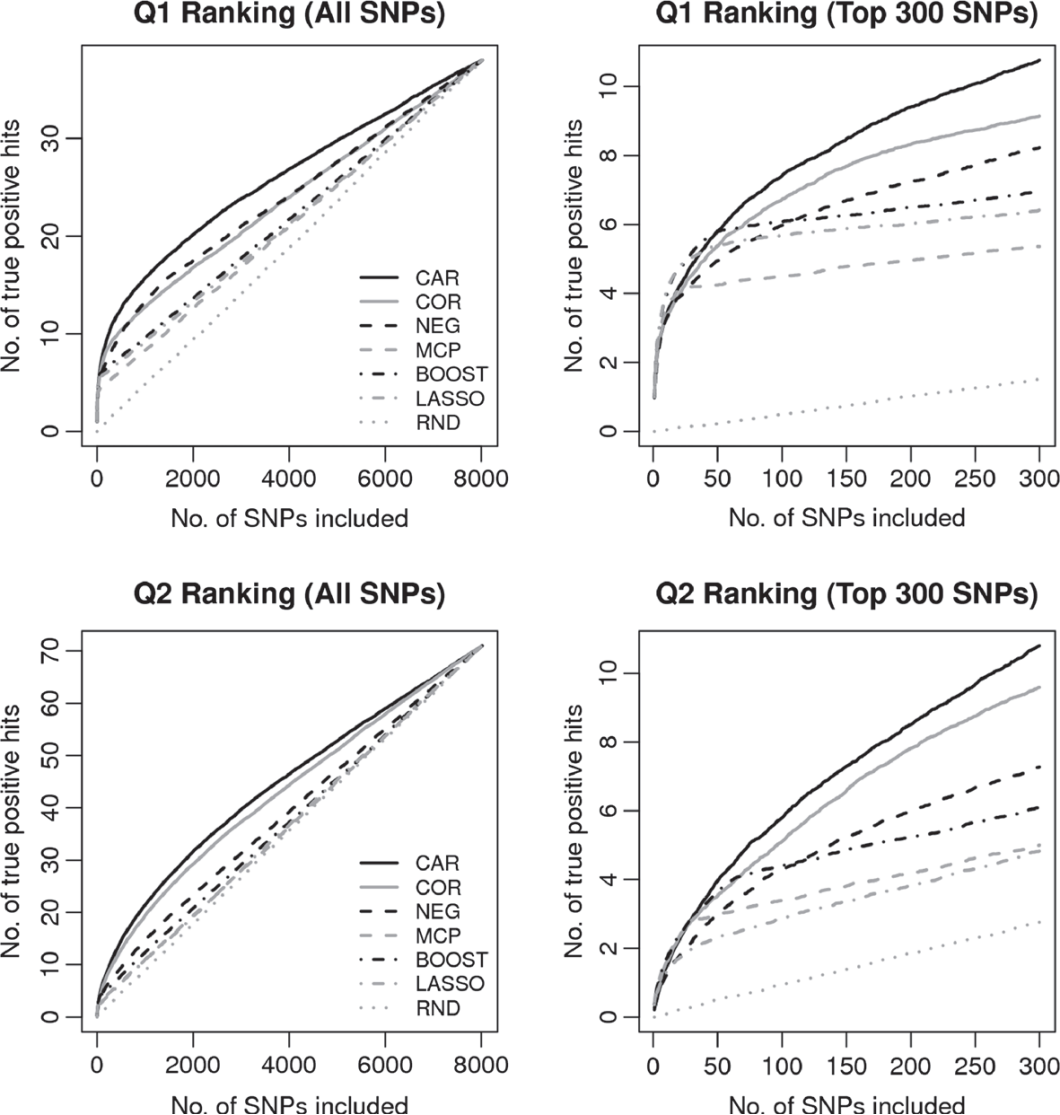
**Average true positives resulting from SNP rankings of the investigated approaches for phenotype Q1 (top row) and Q2 (bottom row).** For Q1 there are 38 true SNPs and for Q2 71 true SNPs.

In Figure 1 and the associated Table 2 we compare the effectiveness of SNP rankings for phenotypes Q1 and Q2. For Q1 all methods uniformly outperform marginal correlation, i.e. at the model size determined by each procedure the number of true positives is larger than that for marginal correlations at the same cutoff. Thus, for Q1 all multivariate SNP selection approaches improve over univariate selection. Moreover, as can be seen from Figure 1 (top row) and Table 2 for small numbers of included SNPs all methods perform similarly but starting from model size of 50 SNPs CAR scores lead to a better ranking in terms of true positives than all other competing approaches. For the more challenging phenotype Q2 the situation is similar. CAR scores almost always provide the most effective ranking (see lower part of Table 2) but intriguingly for this phenotype it is also the only multivariate method that improves over marginal correlation.

**Table 2:** Median model sizes and the corresponding interquartile ranges (IQR) as well as the average true positives for phenotypes Q1 and Q2 for all investigated methods summarized across the 200 repetitions (first three columns)

	**Results**			**Comparisons**		
	**Method**	**Model size**	**TP**	**TP**	**TP**	**TP**
		**Median (IQR)**	**Method**	**CAR**	**COR**	**RND**
Q1						
	CAR	51 (53)	**5.85**	**5.85**	*5.42*	0.23
	COR	176 (108)	*8.06*	**8.99**	*8.06*	0.88
	NEG	1390 (118)	*15.31*	**17.57**	14.38	6.60
	MCP	20 (5)	*4.11*	**4.19**	3.95	0.12
	BOOST	53 (5)	*5.84*	**5.91**	5.50	0.25
	LASSO	37 (31)	*5.19*	**5.21**	4.89	0.18
Q2						
	CAR	31 (38)	**2.93**	**2.93**	*2.85*	0.29
	COR	1 (7)	**0.38**	*0.21*	**0.38**	0.00
	NEG	1632 (755)	20.21	**28.08**	*25.90*	14.50
	MCP	29 (5)	2.75	**2.82**	*2.76*	0.28
	BOOST	59 (6)	*3.92*	**4.34**	3.82	0.59
	LASSO	15 (36)	1.50	*1.88*	**1.97**	0.14

For comparison, the last three columns show the average true positives at the specified model size for CAR, COR and RND. The best performing method is shown in bold, the second best in italic.

In Table 2 we also list the median model sizes for each regression approach. LASSO and MCP generally lead to small numbers of selected SNPs (less than 40), BOOSTING, CAR and COR variable sets are medium sized and NEG chooses a very large number of SNPs. Note the variability in the estimated model sizes as quantified by the corresponding interquartile ranges (IQR) is largest in the methods that estimate the threshold adaptively from the data (CAR, COR, LASSO) whereas it is smallest for those methods where we used a fixed regularization parameter (NEG, MCP, BOOST). Finally, in Table 3 the model sizes and IQR for phenotype Q4 is shown for the investigated methods. Here, COR and LASSO lead to the smallest model sizes and thus the smallest number of false positives, with the MCP and CAR methods being the runners-up.

**Table 3:** Median model sizes and the corresponding interquartile ranges (IQR) for phenotype Q4


Q4							
	Model Size	CAR	COR	NEG	MCP	BOOST	LASSO
	Median	34	0	1900	27	59	1
	IQR	40	1	2713	4	6	6

**Figure 2:**
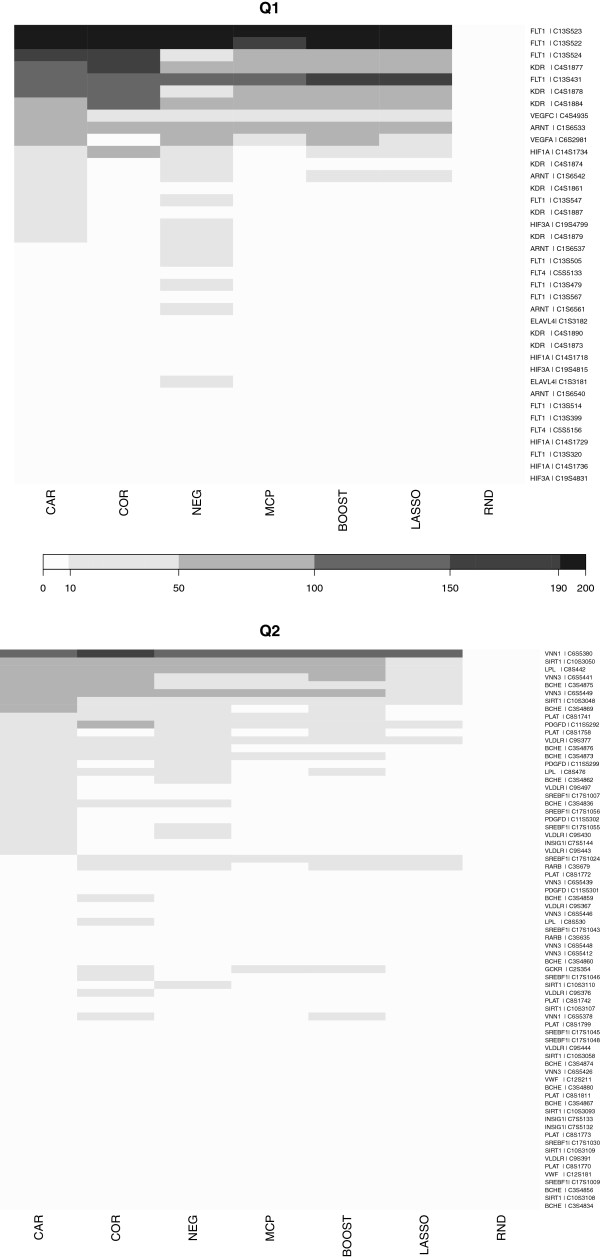
**Frequency of occurrence of each true SNP among the top 100 SNPs selected by each approach for phenotype Q1 (top row) and for Q2 (lower row) for the 200 repetitions.** Note that the SNPs are ordered according to the first column.

In further investigation of these results we identified the actual true SNPs recovered by each SNP selection approach. Specifically, we counted which of the 38 respectively 71 true causal SNPs for Q1 and Q2 were found among the first 100 top ranking SNPs using the 200 repetitions available for each phenotype. The result is shown as a heatmap in Figure 2 and visualizes the relative difficulty of recovering the individual causal SNPs. In Q1, there are two SNPs on top of the heatmap that are consistently detected by all methods. Then, there is a large block primarily recovered by CAR score and correlation, but not by the other approaches. Finally, there are some moderate detections only in CAR scores and NEG regression. Half of the true positives are hardly discovered by any method. The comparison with randomly ordered SNPs (column RND) shows that those SNPs only appear by chance. For Q2, there is only a single SNP that is consistently included in all models. As in Q1, it is followed by a small group of detections most prominent in CAR score and correlation. Finally, there are some moderate findings for both, the CAR score and NEG, and some only for correlation. In addition, hierarchical clustering of the columns (methods) in this heatmap (tree not shown in figure) reveals a basic similarity pattern among the methods: CAR and COR cluster together, NEG and MCP regression form another cluster, and LASSO and BOOST are grouped together.

In Table 4 we list the SNPs identified by the CAR score among the top 100 SNPs in at least 50 of 200 repetitions along with their minor allele frequency (MAF) and BETA values. We consider SNPs with a MAF value smaller than 0.01 as rare and SNPs with a larger MAF value as common variants. The BETA value measures the effect size in the actual simulation of the phenotype [10]. We find large differences between true positive SNPs of the two phenotypes. Whereas Q1 is characterized by SNPs with strong effects and moderate MAFs, the true SNPs for Q2 have a very low MAF and are much harder to detect. Interestingly, most of the SNPs recovered by CAR scores are rare SNPs with comparatively large BETA values. Common SNPs are found as well, then also with small effect values. Thus, CAR scores are successful in achieving a high true positive rate because they not only allow to identify common SNPs but also SNPs with small MAF if a strong signal is present (large BETA).

**Table 4:** True SNPs found among the top 100 SNPs identified by CAR scores in at least 50 of the 200 repetitions for Q1 and Q2

	**SNP**	**Frequency**	**MAF**	**BETA**	**Correlation**
Q1					0.014
	ARNT | C1S6533	88	0.011478	0.56190	
	FLT1 | C13S431	110	0.017217	0.74136	0.147
	FLT1 | C13S522	200	0.027977	0.61830	0.147
	FLT1 | C13S523	200	0.066714	0.64997	0.147
	FLT1 | C13S524	164	0.004304	0.62223	0.147
	KDR | C4S1877	145	0.000717	1.07706	0.111
	KDR | C4S1878	101	0.164993	0.13573	0.111
	KDR | C4S1884	95	0.020803	0.29558	0.111
	VEGFA | C6S2981	69	0.002152	1.20645	
	VEGFC | C4S4935	91	0.000717	1.35726	
Q2					0.008
	BCHE | C3S4869	54	0.000717	1.01569	0.001
	BCHE | C3S4875	59	0.000717	1.09484	0.001
	LPL | C8S442	69	0.015782	0.49459	
	SIRT1 | C10S3048	54	0.002152	0.83224	0.330
	SIRT1 | C10S3050	72	0.002152	0.97060	0.330
	VNN1 | C6S5380	138	0.170732	0.24437	
	VNN3 | C6S5441	59	0.098278	0.27053	0.066
	VNN3 | C6S5449	57	0.010043	0.66909	0.066

The last column shows the average absolute correlation among all SNPs for Q1 and Q2 as well as the average absolute correlation for the SNPs belonging to one gene.

The last column in Table 4 provides information about the average absolute correlation among all true SNPs for Q1 and Q2 as well as among the identified SNPs on the same gene. We observe that the true positive SNPs in Q1 best identified by the CAR score are highly correlated within the same gene. This demonstrates that the CAR score successfully utilizes the correlation structure among SNPs to optimize the ranking. For phenotype Q2 the correlation among the true SNPs is generally lower compared to Q1, still except for BCHE the correlation among SNPs on the same gene is larger compared to the average correlation between a randomly chosen pair of true SNPs.

Finally, in Table 5 we provide the proportion of rare and common SNPs found among the top ranking 100 SNPs for each methods. This also shows that the proposed approach based on CAR scores is effective in finding rare SNPs.

**Table 5:** Proportion of common and rare variants of the true SNPs found among the top 100 SNPs


Q1							
	Proportion (%)	CAR	COR	NEG	MCP	BOOST	LASSO
	Common	0.56	0.71	0.63	0.74	0.71	0.73
	Rare	0.44	0.29	0.37	0.26	0.29	0.27
Q2							
	Proportion (%)	CAR	COR	NEG	MCP	BOOST	LASSO
	Common	0.28	0.41	0.36	0.44	0.42	0.43
	Rare	0.72	0.59	0.64	0.56	0.58	0.57

## Conclusions

Large scale simultaneous SNP selection is a statistically and computationally very challenging task. To this end, we have introduced here a novel algorithm based on CAR score regression that can be applied effectively in high dimensions. Subsequently, in a comparison study we have investigated five multivariate regression-based SNP selection approaches with regard to their ability to correctly recover causal SNPs and corresponding SNP rankings.

As overall best method we recommend using CAR scores since this method was the only approach not only consistently outperforming the competing other multivariate SNP selection procedures in terms of identified true positives but also the only approach uniformly improving over simple univariate ranking by marginal correlation. In addition we have shown that CAR scores also are successful in detecting rare variants which recently have been recognize to be important indicators for human disease [29,30].

## Competing interests

The authors declare that they have no competing interests.

## Authors’ contributions

VZ, PDS, and KS jointly developed the algorithm. VZ performed the analyzes. VZ and KS wrote the manuscript. All authors read and approved the manuscript.

## References

[B1] Hoggart CJ Whittaker JC De Iorio M Balding DJ Analysis of all SNPs, in genome-wide and re-sequencing association studies PLoS Genetics 2008 4 e1000130 10.1371/journal.pgen.1000130 18654633 PMC2464715

[B2] Ayers KL Cordell H SNP selection in genome-wide and candidate gene studies via penalized logistic regression Genet Epidemiol 2010 34 879 891 10.1002/gepi.20543 21104890 PMC3410531

[B3] Guan Y Stephens M Bayesian variable selection regression for genome-wide association studies, and other large-scale problems Ann Appl Statist 2011 5 1780 1815 10.1214/11-AOAS455

[B4] Armitage P Tests for linear trends in proportions and frequencies Biometrics 1955 11 375 386 10.2307/3001775

[B5] Foulkes AS Applied Statistical Genetics with R 2009 New York: Springer

[B6] Wu TT Chen YF Hastie T Sobel E Lange K Genome-wide association analysis by lasso penalized logistic regression Bioinformatics 2009 25 714 721 10.1093/bioinformatics/btp041 19176549 PMC2732298

[B7] Hothorn T Bühlmann P Model-based boosting in high dimensions Bioinformatics 2006 22 2828 2829 10.1093/bioinformatics/btl462 16940323

[B8] Zuber V Strimmer K Gene ranking and biomarker discovery under correlation Bioinformatics 2009 25 2700 2707 10.1093/bioinformatics/btp460 19648135

[B9] Zuber V Strimmer K High-dimensional regression and variable selection using CAR scores Statist Appl Genet Mol Biol 2011 10 34

[B10] Almasy L Dyer TD Peralta JM Kent Jr JW Charlesworth JC Curran JE Blangero J Genetic analysis workshop 17 mini-exome simulation BMC Proceedings 2011 5 Suppl 9 S2 10.1186/1753-6561-5-S9-S2 22373155 PMC3287854

[B11] Efron B Empirical Bayes, estimates for large-scale prediction problems J Amer Statist Assoc 2009 104 1015 1028 10.1198/jasa.2009.tm08523 PMC2844005 20333278

[B12] Fan J Lv J Sure independence screening for ultra-high dimensional feature space (with discussion) J R Statist Soc B 2008 70 849 911 10.1111/j.1467-9868.2008.00674.x PMC2709408 19603084

[B13] Ardlie KG Kruglyak L Seielstad M Patterns of linkage disequilibrium in the human genome Nat Rev Genet 2002 3 299 309 10.1038/nrg777 11967554

[B14] Klaus B Strimmer K Signal identification for rare and weak features: higher criticism or false discovery rates? Biostatistics 2012 in press 10.1093/biostatistics/kxs030 22962499

[B15] Allen GI Tibshirani R Inference with transposable data: modelling the effects of row and column correlations J R Statist Soc B 2012 74 721 743 10.1111/j.1467-9868.2011.01027.x PMC8649963 34880705

[B16] Schäfer J Strimmer K A shrinkage approach to large-scale covariance matrix estimation and implications for functional genomics Statist Appl Genet Mol Biol 2005 4 32 10.2202/1544-6115.1175 16646851

[B17] Hastie T Tibshirani T Efficient quadratic regularization for expression arrays Biostatistics 2004 5 329 340 10.1093/biostatistics/kxh010 15208198

[B18] R Development Core Team R: A Language and Environment for Statistical Computing 2012 Vienna: R Foundation for Statistical Computing [ http://www.R-project.org]. [ISBN 3-900051-07-0].

[B19] Zhang CH Nearly unbiased variable selection under minimax concave penalty Ann Statist 2010 38 894 942 10.1214/09-AOS729

[B20] Schapire RE The strength of weak learnability Machine Learning 1990 5 197 227

[B21] Tibshirani R Regression shrinkage and selection via the lasso J R Statist Soc B 1996 58 267 288

[B22] Breheny P Huang J Coordinate descent algorithms for nonconvex penalized regression, with applications to biological feature selection Ann Applied Statistics 2011 5 232 253 10.1214/10-AOAS388 PMC3212875 22081779

[B23] Friedman J Hastie T Tibshirani R Regularization paths for generalized linear models via coordinate descent J Statist Soft 2010 39 1 13 PMC2929880 20808728

[B24] Donoho D Jin J Higher criticism thresholding: optimal feature selection when useful features are rare and weak Proc Natl Acad Sci USA 2008 105 14790 15795 10.1073/pnas.0807471105 18815365 PMC2553037

[B25] Donoho D Jin J Feature selection by higher criticism thresholding achieves the optimal phase diagram Phil Trans R Soc A 2009 367 4449 4470 10.1098/rsta.2009.0129 19805453

[B26] Duarte Silva AP Two-group classification with high-dimensional correlated data: a factor model approach Comput Stat Data An 2011 55 2975 2990 10.1016/j.csda.2011.05.002

[B27] Strimmer K fdrtool: a versatile R package for estimating local and tail area-based false discovery rates Bioinformatics 2008 24 1461 1462 10.1093/bioinformatics/btn209 18441000

[B28] Strimmer K A unified approach to false discovery rate estimation BMC Bioinformatics 2008 9 303 10.1186/1471-2105-9-303 18613966 PMC2475539

[B29] Bodmer W Bonilla C Common and rare variants in multifactorial susceptibility to common diseases Nat Genet 2008 40 695 701 10.1038/ng.f.136 18509313 PMC2527050

[B30] McClellan J King MC Genetic heterogeneity in human disease Cell 2010 141 210 217 10.1016/j.cell.2010.03.032 20403315

